# Identification of Genetic Modifiers of TDP-43: Inflammatory Activation of Astrocytes for Neuroinflammation

**DOI:** 10.3390/cells10030676

**Published:** 2021-03-18

**Authors:** Jae-Hong Kim, Md Habibur Rahman, Donghwi Park, Myungjin Jo, Hyung-Jun Kim, Kyoungho Suk

**Affiliations:** 1 Department of Pharmacology, School of Medicine, Kyungpook National University, Daegu 41944, Korea; kim86217@nate.com (J.-H.K.); habib.edu.bd@gmail.com (M.H.R.); bdome@hanmail.net (D.P.); jinyhoi11@naver.com (M.J.); 2 BK21 Plus KNU Biomedical Convergence Program, Department of Biomedical Sciences, School of Medicine, Kyungpook National University, Daegu 412944, Korea; 3 Brain Science & Engineering Institute, Kyungpook National University, Daegu 41566, Korea; 4 Dementia Research Group, Korea Brain Research Institute (KBRI), Daegu 41062, Korea; kijang1@kbri.re.kr

**Keywords:** TDP-43, astrocyte, glia, neuroinflammation, genetic interaction, amyotrophic lateral sclerosis

## Abstract

Transactive response DNA-binding protein 43 (TDP-43) is a ubiquitously expressed DNA/RNA-binding protein linked to amyotrophic lateral sclerosis (ALS) and frontotemporal dementia (FTD). TDP-43 has been implicated in numerous aspects of the mRNA life cycle, as well as in cell toxicity and neuroinflammation. In this study, we used the toxicity of the TDP-43 expression in *Saccharomyces cerevisiae* as an assay to identify *TDP-43* genetic interactions. Specifically, we transformed human *TDP-43* cDNAs of wild-type or disease-associated mutants (*M337V* and *Q331K*) en masse into 4653 homozygous diploid yeast deletion mutants and then used next-generation sequencing readouts of growth to identify yeast toxicity modifiers. Genetic interaction analysis provided a global view of TDP-43 pathways, some of which are known to be involved in cellular metabolic processes. Selected putative loci with the potential of genetic interactions with *TDP-43* were assessed for associations with neurotoxicity and inflammatory activation of astrocytes. The pharmacological inhibition of succinate dehydrogenase flavoprotein subunit A (SDHA) and voltage-dependent anion-selective channel 3 (VDAC3) suppressed TDP-43-induced expression of proinflammatory cytokines in astrocytes, indicating the critical roles played by SDHA and VDAC3 in TDP-43 pathways during inflammatory activation of astrocytes and neuroinflammation. Thus, the findings of our *TDP-43* genetic interaction screen provide a global landscape of TDP-43 pathways and may help improve our understanding of the roles of glia and neuroinflammation in ALS and FTD pathogenesis.

## 1. Introduction

Transactive response DNA-binding protein 43 kDa (TDP-43), a DNA/RNA-binding protein encoded by the *TARDBP* gene in humans, is involved in transcriptional repression and splicing and stability of RNA [1]. It is a heterogeneous nuclear ribonucleo-type protein ubiquitously expressed in eukaryotic cells, particularly in the nucleus [2]. Inclusions of wild-type (WT) and missense mutations of TDP-43 are major causes of amyotrophic lateral sclerosis (ALS) pathology [3]. In several studies, cellular overexpression of TDP-43 has been reported to cause TDP-43 truncation, increased cytoplasmic and nuclear ubiquitin levels, and intranuclear and cytoplasmic aggregates associated with ALS pathology [3]. Similarly, more than 50 missense mutations in the *TDP-43* gene have been associated with familial and sporadic cases of ALS [4]. Among them, M337V and Q331K are well-studied mutations for which several ALS-disease models have been established [5]. In this study, we built upon these findings to compare the characteristics of WT TDP-43 and these ALS-associated mutants in terms of genetic interactions and relevant signaling pathways.

Cytoplasmic accumulation of TDP-43 in motor neurons is one of the major features of ALS and frontotemporal dementia (FTD). However, recent studies have demonstrated that cytoplasmic inclusions of TDP-43 are not only restricted to motor neurons but also found in glial cells, particularly astrocytes [6]. TDP-43 inclusions in reactive astrocytes are sufficient to cause motor neuron death and present auto-cytotoxicity in rodent models of ALS [7,8,9,10]. Thus, reactive astrocytes have been suggested to play a vital role in ALS-disease progression and may be the primary driver of TDP-43-mediated proteinopathies. Astrocytes are abundant and heterogeneous homeostatic cells of the central nervous system (CNS), in which they provide metabolic and physical support to neurons [11]. In response to any CNS insults, such as injury and disease, astrocytes undergo a morphological, biochemical, transcriptional, and functional transformation known as astrogliosis or astrocyte reactivity [12,13,14]. This inflammatory phenotypic change in astrocytes is characterized by increased expression of several proinflammatory molecule types, such as cytokines and chemokines, along with a hypertrophic morphology and thick processes relative to nonreactive astrocytes [15,16]. Such reactive astrocytes are thought to be involved in the propagation of inflammatory neurodegeneration [17].

Inflammatory activation of astrocytes is a well-accepted feature of animal models of ALS, such as superoxide dismutase-1 (SOD1) mutant–mediated ALS [18,19]. In the spinal cords of loxSOD1G37R mice, an elevated proportion of glial fibrillary acidic protein (GFAP)-positive astrocytes is observed before disease onset [20]. This astrogliosis is rapidly apparent at the onset, progressive, and more prominent during disease progression; however, the exact percentage of astrocytes that show inflammatory action at onset is yet to be reported. Moreover, microglial activation and the release of inflammatory factors have been reported to occur at the earliest point of disease onset and to be involved in the propagation of neurotoxic reactive astrocytes during disease progression [20]. The inflammatory activation of astrocytes is most prevalent in animal models of TDP-43 proteinopathy [21]. To develop therapeutic interventions for neuroinflammatory and degenerative diseases, it is necessary to identify downstream signaling pathways of TDP-43 in reactive astrocytes. Lee et al. recently revealed that TDP-43 overexpression in astrocytes causes neurodegeneration through a protein tyrosine phosphatase 1B (PTP1B)-mediated inflammatory response characterized by increased levels of inflammatory molecules, such as interleukin-1β (IL-1β), tumor necrosis factor-α (TNF-α), interleukin-6 (IL-6), lipocalin-2 (LCN2), and inducible nitric oxide synthase (iNOS) [22]. They also found that PTP1B regulates the inflammatory response via activation of the nuclear factor-κB (NF-κB) pathway, accompanied by increased phosphorylation of NF-κB subunit p65 in astrocytes. Ser536 phosphorylation of p65 is required for nuclear translocation of NF-κB, which in turn induces expression of inflammatory genes [23]. NF-κB p65 (Ser536) phosphorylation was significantly increased in *TDP-43*-transfected astrocytes. Nevertheless, the downstream pathways directly regulated by TDP-43 in reactive astrocytes have yet to be further investigated.

Previous studies have suggested that mitochondrial dysfunction is a critical factor for many neurodegenerative diseases, including Alzheimer’s disease (AD), Parkinson’s disease (PD), and ALS [24,25,26]. Moreover, TDP-43 is linked to abnormalities of mitochondrial structure and function [27,28]. Therefore, TDP-43-induced mitochondrial defects could be a key feature of disease pathology. Also, the mitochondrial dysfunction in glial cells has been characterized by decreased adenosine triphosphate (ATP) levels, loss of mitochondrial inner membrane polarization, and increased mitochondrial reactive oxygen species (ROS) production, which has been correlated with astrocyte activation toward the A1 inflammatory state [11]. However, the role of *TDP-43*-interacting genetic loci in mitochondrial dysfunction and inflammatory activation of astrocytes is not clearly understood.

The objective of this study is to better understand the TDP-43 signaling pathways and to uncover new drug targets related to these pathways. In this study, we sought to exploit a model eukaryote which is amenable to the rapid genome-scale experimentation approach to identify related functions. More specifically, we performed a genetic interaction screen for yeast deletions that can relieve the toxic effects of TDP-43 expression in yeast, thereby identifying orthologous human genes as candidate genetic interaction loci. Genetic interaction analysis revealed many *TDP-43*-interacting genetic loci, including succinate dehydrogenase A (*SDHA*), heat shock protein 90 alpha family class B member 1 (*HSP90AB1*), and voltage-dependent anion channel 3 (*VDAC3*). Subsequent cultured astrocyte-based validation experiments of these candidates identified key determinants of neuroinflammation and mitochondrial dysfunction, helping to elucidate the molecular mechanisms of TDP-43-mediated pathogenesis.

## 2. Materials and Methods

### 2.1. Yeast Strains, Media, and Plasmids

BY4742 (Mat α; his3Δ1; leu2Δ0, lys2Δ0; ura3Δ0) was used as the WT yeast strain in this study. The Homozygous Diploid Complete Set of Yeast Deletion Clones and Homozygous Diploid Yeast Deletion Pools were purchased from Invitrogen (Carlsbad, CA, USA). Yeast cells were grown in rich medium or synthetic medium lacking leucine but containing 2% glucose (SD-Leu), raffinose (SRaf-Leu), or galactose (SGal-Leu). WT and ALS-linked mutant (*M337V* and *Q331K*) *TDP-43* cDNAs were kindly provided by Prof. Hyung-Jun Kim at Korea Brain Research Institute (KBRI) (Daegu, Republic of Korea) [22]. For yeast expression, the Gateway LR reaction between an attL-containing entry clone and an attR-containing destination vector to generate an expression clone was used to shuttle *TDP-43* cDNA into *pAG425GAL-ccdB* (Addgene, Cambridge, MA, USA) [29]. All plasmids were 2 μm-based and under the control of the *GAL1* promoter. All constructs were verified by Sanger sequencing. *TDP-43* cDNAs in *pAG425GAL* (the yeast destination vector) were transformed into BY4742 or homozygous diploid deletion strains. All yeast strains were grown at 30 °C according to the standard protocol. We used the LiAc/SS carrier DNA/ polyethylene glycol (PEG) method to transform yeast with plasmid DNA, as previously described [30]. Because the green fluorescence protein (GFP)-fused WT and mutant *TDP-43* are under the control of the same promoter in the same plasmid, similar levels of expression were observed in the transformed yeast, as represented by GFP intensity (Supplementary Figure S1). For functional studies in mammalian cells, the Gateway LR reaction was used to shuttle *TDP-43* cDNA into *pDS-GFP-XB* (Invitrogen) destination vectors.

### 2.2. TDP-43 Genetic Interaction Screen

The screen for genetic modifiers of *TDP-43* in *Saccharomyces cerevisiae* was conducted as previously described [31,32,33]. Briefly, WT and mutant (*M337V* and *Q331K*) human *TDP-43* cDNAs were transformed into homozygous diploid yeast deletion pools containing 4653 individual deletion clones. The transformants were then incubated in SD-Leu medium for 16 h. All transformants containing either WT or mutant forms of *TDP-43* are isogenic because *TDP-43* constructs are maintained episomally rather than being integrated into yeast chromosomes. The cells were washed twice with Phosphate-buffered saline (PBS) and then incubated in the SGal-Leu medium for two days. Cells remaining in the glucose-containing SD-Leu medium were used as a control. Genomic DNA was isolated from cells that were harvested after pooled growth. Each 20-mer upstream barcode was amplified and sequenced using a Genome Analyzer (Illumina, San Diego, CA, USA) according to the manufacturer’s protocols, as previously described [32,33]. In brief, the barcode was amplified using composite primers: 5′-GATGTCCACGAGGTCTCT-3′ (forward) and 5′-GTCGACCTGCAGCGTACG-3′ (reverse). Barcode sequencing data were analyzed by rescaling and converting into Z-scores for each yeast deletion gene [32,33]. Toxicity-suppressing genetic interactions were identified on the basis of Z-scores > 1.96. The critical Z-score values with 95% confidence level are −1.96 and +1.96 standard deviations (SD). The uncorrected *p*-value associated with the 95% confidence level is 0.05.

### 2.3. Constructing a Genetic Interaction Network

The GeneMania plugin from Cytoscape was used to build the interaction network for *TDP-43* and its genetic interaction loci [34,35]. Initially, three distinct subnetworks for each form of *TDP-43* (*WT*, *M337V* mutant, and *Q331K* mutant) were constructed. These subnetworks were then merged with common interacting gene nodes in the Cytoscape environment [35]. The tangential nodes were manually deleted; thus, only highly connected nodes were included for further analysis. The predicted co-expressions, co-localizations, and genetic interactions derived from the GeneMania interaction network were also included for enrichment analysis [34]. The functional annotation-clustering tool of the online bioinformatics resource Database for Annotation, Visualization, and Integrated Discovery (DAVID) version 6.8 [36] was used to perform Gene Ontology (GO) analysis limited to biological process (BP) terms. When they satisfied a false discovery rate limit of 10%, GO terms were considered enriched.

### 2.4. Cell Cultures, Transfection, and Reagents

N2a mouse neuroblastoma cells were kindly provided by Prof. Hyung-Jun Kim (Dementia Research Group, KBRI, Daegu, Republic of Korea). The cell line was authenticated, tested for mycoplasma (with a negative result), and maintained in Dulbecco’s modified Eagle’s medium (DMEM) supplemented with 5% heat-inactivated fetal bovine serum (FBS) and 100 U/mL penicillin at 37 °C. Primary astrocyte cultures were prepared as previously described [37]. In brief, the whole brains of 3-day-old C57BL/6 mice were homogenized and mechanically disrupted using a nylon mesh. The obtained mixed glial cells were seeded in culture flasks and cultured in DMEM supplemented with 10% FBS, 100 U/mL penicillin, and 100 μg/mL streptomycin at 37 °C in a 5% CO_2_ incubator. The culture media were first changed after five days and then subsequently every three days. After 14 days of culture, primary astrocytes were obtained from mixed glial cells using a mechanical shaker (200 rpm for 12 h).

For transient transfection, cells were transfected with *pCMV6-AC-GFP* containing WT or mutant (*M337V* and *Q331K*) human *TDP-43* cDNA using Lipofectamine 2000 transfection reagent (Thermo Fisher Scientific, Waltham, MA, USA) following the manufacturer’s instructions. The transient transfectants were established according to their GFP expression as observed under a fluorescence microscope (BX50; Olympus, Tokyo, Japan), and transfected cells were subjected to fluorescence-activated cell sorting (FACS). GFP-expressing cells were treated with a pharmacological inhibitor or dimethyl sulfoxide (DMSO) for 24 h. Malonate (Cat No. M4795) was purchased from Sigma-Aldrich (St. Louis, MO, USA), geldanamycin (Cat No. BML-EI280) from Enzo Life Science (Farmingdale, NY, USA), and TRO19622 (Cat No. 2906) from Tocris Bioscience (Bristol, UK).

### 2.5. Cell Viability Assays

For the 3-[4, 5-dimethylthiazol-2-yl]-2, 5-diphenyltetrazolium bromide (MTT) assay, cells were treated with various reagents for a designated period. After treatment, MTT (0.5 mg/mL; Sigma-Aldrich) was added to the cells, which were then incubated for 2 h at 37 °C in a 5% CO_2_ incubator. Subsequently, dimethyl sulfoxide (DMSO) was added to dissolve the insoluble crystals and the absorbance was measured at 570 nm using a microplate reader (Anthos Labtec Instruments, Wals, Austria).

### 2.6. Measurement of Mitochondrial Reactive Oxygen Species

Mitochondrial reactive oxygen species (ROS) levels were measured as previously described [38]. GFP-transfected cells were sorted and seeded in 96-well plates, and then treated with a pharmacological inhibitor for 24 h. Afterward, they were rinsed in PBS and stained with mitochondrial-superoxide indicator (MitoSOX-Red) for 20 min at 37 °C. After washing 2× with PBS, cells were fixed with 4% paraformaldehyde for 20 min at room temperature. MitoSOX-Red fluorescence was imaged using a Lionheart FX-automated microscope (BioTek, Winooski, VT, USA) and the average fluorescence intensity was calculated. Fluorescence was measured with an excitation and emission of 549 and 575 nm, respectively (Texas-Red filter). To account for variations in cell location in the well, fluorescence was measured with a 2 × 2 area scan and the results were averaged. The mean objective intensity was measured and normalized by counting the cells in wells using Gen5TM 3.0 software (BioTek, Winooski, VT, USA).

### 2.7. Measurement of Mitochondrial Membrane Potentiation

Mitochondrial membrane potential in live cells was assessed using the tetramethylrhodamine ethyl ester perchlorate (TMRE) (Thermo Fisher Scientific, Waltham, MA, USA) probe as described previously [39]. For live imaging, GFP-transfected cells were sorted and seeded in 96-well plates and then treated with 200 nM TMRE for 20 min at 37 °C, before being treated with pharmacological inhibitors in DMSO. The cells were then imaged, and TMRE-stained cells were quantified with a Lionheart FX-automated microscope (Biotek, Winooski, VT, USA). Fluorescence was measured and averaged and mean objective intensity was measured and normalized, as described in Section 2.6.

### 2.8. Real-Time Polymerase Chain Reaction

RNA was extracted from cells using TRIzol reagent (Thermo Fisher Scientific, Waltham, MA, USA), and RNA was “cleaned up” using a RNeasy Mini Kit (QIAGEN, Hilden, Germany) according to the manufacturer’s instructions. Using 100 ng of RNA, cDNA was synthesized at 37 °C for 120 min with a High-Capacity cDNA Reverse Transcription Kit (Thermo Fisher Scientific). Quantitative real-time polymerase chain reaction (RT-PCR) [40] was performed using a One-Step SYBR PrimeScript RT-PCR Kit (Perfect Real Time; Takara Bio Inc.) according to the manufacturer’s instructions, and detection was then conducted using an Applied Biosystems 7500 Real-Time PCR system (Applied Biosystems). RT-PCR was performed using two reference genes (glyceraldehyde 3-phosphate dehydrogenase (*Gapdh)* and β-actin *(Actb)*) for the quantification of gene expression, as previously described [41,42]. The 2^−ΔΔCt^ method was used to calculate the relative differences in gene expression [40]. The primers used in quantitative PCR analyses of mouse *Tnf*, *Il1b*, *Gapdh*, and *Actb* were as follows: *Tnf*: 5′-CAT CTT CTC AAA ATT CGA GTG ACA A-3′ (forward), 5′-ACT TGG GCA GAT TGA CCT CAG-3′ (reverse); *Il1b*: 5′-AGT TGC CTT CTT GGG ACT GA-3′ (forward), 5′-TCC ACG ATT TCC CAG AGA AC-3′ (reverse); *Gapdh*: 5′-TGG GCT ACA CTH AHC ACC AG-3′ (forward), 5′-GGG TGT CGC TGT TGA AGT CA-3′ (reverse); *Actb*: 5′-ATC CGT AAA GAC CTC TAT GC-3′ (forward), 5′-AAC GCA GCT CAG TAA CAG TC-3′ (reverse).

### 2.9. Enzyme-Linked Immunosorbent Assay

The levels of IL-1β and TNF-α in culture media were quantified using an enzyme-linked immunosorbent assay (ELISA) kit (R&D Systems, Minneapolis, MN, USA). ELISA assays were conducted in 96-well plates using the media and methods stipulated in the manufacturer’s instructions. For the standards, mouse recombinant proteins were used at concentrations of 10–2500 pg/mL. IL-1β and TNF-α protein levels were normalized to the total protein content of the astrocyte culture media samples. All measurements were obtained from duplicated assays.

### 2.10. Western Blot Analysis

Cultured cells were lysed in a triple-detergent lysis buffer (50 mM Tris-HCl (pH 8), 150 mM NaCl, 0.02% sodium azide, 0.1% NaDodSO4 (SDS), 1% Nonidet P-40, 0.5% sodium deoxycholate, and 1 mM phenylmethylsulfonyl fluoride (PMSF)). Protein concentrations in cell lysates were determined using a protein assay kit (Bio-Rad, Hercules, CA, USA). Each protein sample was separated by 12% sodium dodecyl sulfate-polyacrylamide gel electrophoresis (SDS-PAGE), before being blotted and incubated overnight at 4 °C using the following primary antibodies: anti-TDP-43 (Proteintech, Rosemont, IL, USA; 10782-2-AP) and anti-β-actin (Thermo Fisher Scientific; MAS-15739). Afterward, blots were incubated with horseradish peroxidase (HRP)-conjugated secondary antibodies and detected using enhanced chemiluminescent (ECL) solution.

### 2.11. Statistical Analysis

Two independent transformants were tested in the yeast spotting assays. Cell viability experiments were performed in eight sister wells (biological replicates), which were not repeated. The number of sister wells was also described in other experiments. Different treatments were compared with Student’s t-test or two-way analysis of variance (ANOVA) with Dunnett’s multiple comparisons test using SPSS software (version 18.0; SPSS Inc., Chicago, IL, USA). *p*-values < 0.05 were considered statistically significant. Sample sizes for experiments were chosen to ensure adequate statistical power on the basis of G*power 3.1 software [43].

## 3. Results

### 3.1. Identification of TDP-43 Genetic Interactions

**Figure 1:**
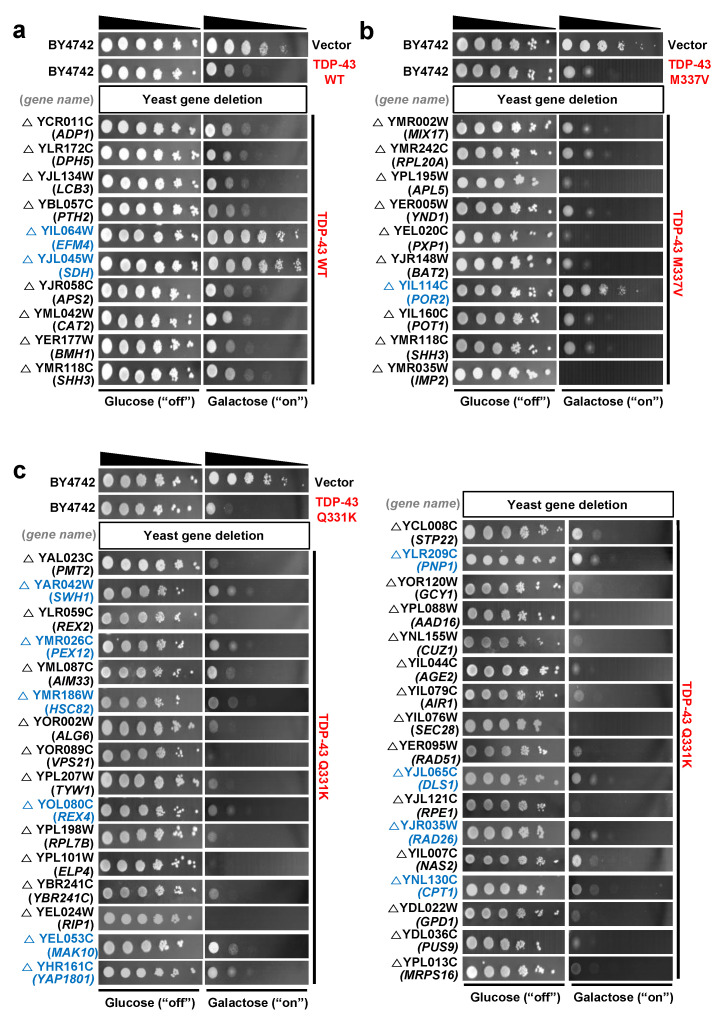
Validation of toxicity suppressors by yeast spotting assays. Based on the analysis of Bar-seq data and Z-score distributions, the toxicity-suppressing yeast gene deletions were subjected to spotting assays. (**a**–**c**) Overexpression of wild-type (WT) (**a**) or mutant human *TDP-43* (**b**,**c**) causes toxicity in yeast. Deletion strains marked in blue show toxicity-suppressing effects. The *pAG425GAL-ccdB* yeast destination vector was used as the control. The BY4742 yeast strain was used as the WT control.

Using the toxicity of TDP-43 overexpression in *S. cerevisiae* as an assay to identify TDP-43 genetic interactions, we found that overexpression of human TDP-43 cDNAs, WT, or disease-associated mutants (M337V and Q331K), caused toxicity in *S. cerevisiae*, as indicated by a lower number of visible spots when compared with the control vector transformation (Figure 1a–c).

In a genome-wide pooled screen to identify genetic interactions (based on toxicity modification as previously described [31,32]), the TDP-43 gene or its two mutant variants were first introduced into a pool of 4653 yeast homozygous deletion strains containing a 20 bp DNA barcode sequence, such that each deletion strain harbored unique barcode sequences next to the deletion locus [33,44]. Afterwards, expression of the TDP-43 WT and mutant genes were induced by yeast growth on galactose media. Each deletion pool expressing TDP-43 or mutant genes was cultured to amplify the yeast barcodes: genomic DNA was isolated from yeast culture and subjected to PCR amplification with the common primers flanking the barcode [33]. Finally, the amplified barcodes were subjected to next-generation sequencing (Bar-seq) to quantify yeast barcode abundances and identify fitness values of TDP-43 genetic interactions [31,32,33,45] (Supplementary Table S1).

The relative abundance of each yeast barcode is a proxy for the differential growth of the corresponding deletion strain, which is based on a positive correlation between the barcode number and growth of a specific yeast strain—higher barcode numbers show higher growth in a specific yeast strain [46]. This system enables identification of TDP-43 toxicity levels in the absence of a specific yeast gene. TDP-43 genetic interactions were identified based on Z-scores, and Z-scores > 1.96 were considered indicative of toxicity suppressors (Table 1).

One overlapping gene (SDHC) was identified as a toxicity suppressor between TDP-43 WT and M337V (Table 1), whereas no overlapping genes were identified as toxicity suppressors between TDP-43 WT and Q331K. We compared the overlap of the genes identified here with those identified in previously published studies. We identified 52 toxicity suppressor genes (Z-score > 1.96; Table 1), among which no genes overlapped with the previous yeast TDP-43 toxicity modifier screen studies by Elden et al. and Kim et al. [47,48], two genes overlapped with the TDP-43 interacting protein study by Freibaum et al. [49], and no genes overlapped with a protein interactome study by Blokhuis et al. [50] (Supplementary Table S2). We also compared the overlap of genes identified in this study with those identified in a previous cross-linking and immunoprecipitation followed by deep sequencing (CLIP-seq) screen study [51,52,53]. Among the 52 toxicity-suppressor genes identified here, four overlapped with previous RNA targets of TDP-43 [52,53] (Supplementary Table S2). This suggests that at least four genetic interaction loci of TDP-43 may reflect the RNA-binding characteristics of TDP-43 (Supplementary Table S2). 

**Figure 2:**
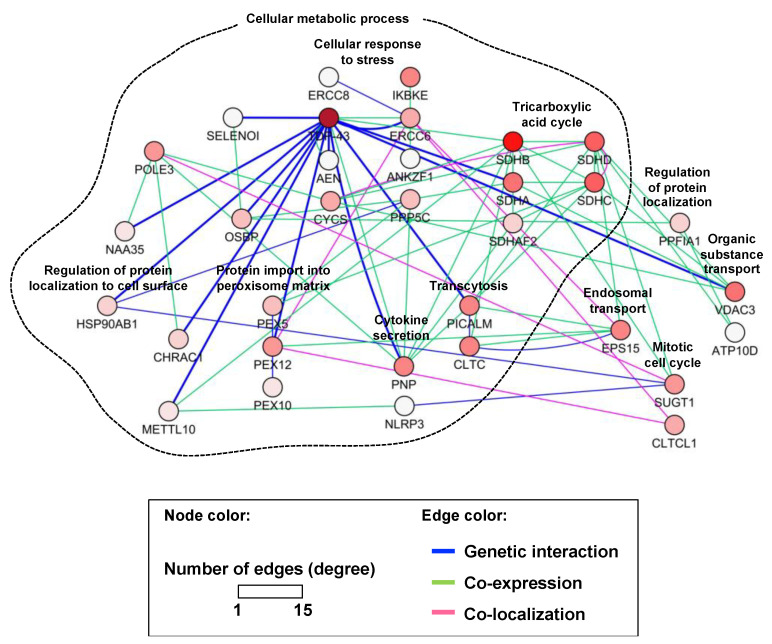
Interaction network of *TDP-43*. Human orthologs were identified for 13 yeast genes, the deletions of which suppressed the toxicity of TDP-43. A network view of the genetic interactions between *TDP-43* and its interaction loci was generated using the GeneMANIA Cytoscape plugin. Other interacting or associated genes were also included in the network. *TDP-43* genetic interactions are highlighted as thick blue lines (edges), and nodes are annotated according to biological processes.

In the yeast validation assay, we found 13 yeast gene deletions that had suppressing effects on TDP-43 toxicity (Figure 1) based on Z-scores (Supplementary Table S1)—higher Z-scores (cut-off value: 1.96) indicated higher suppressing effects. Human orthologs were then identified for these 13 yeast genes using the Karolinska Institute’s InParanoid Database v8.0 (http://inparanoid.sbc.su.se, accessed on January 2017) [54], and these orthologs were subjected to in silico prediction analysis for the possible gene association network using the GeneMANIA Cytoscape plugin v3.5.2 (http://genemania.org, accessed on December 2020) [55]. A TDP-43 network was constructed based on the genetic interaction of TDP-43 with the 13 interacting loci and other predicted genes (Figure 2).

Co-expression indicates when two genes are linked if their expression levels are similar across conditions or they are expressed together in a gene expression study. Similarly, two genes are defined to be co-localized if they are both expressed in the same tissue or if their gene products are both identified in the same cellular location. The GeneMania plugin from Cytoscape was used to build the interaction network for common pathways that might regulate such functional interactions [34,35].

According to the DAVID analysis, the nodes of the network were enriched for several functional groups (biological processes) as follows: tricarboxylic acid cycle, protein import into peroxisome matrix, cellular metabolic process, endosomal transport, organic substance transport, transcytosis, regulation of protein localization to cell surface, cytokine secretion, regulation of protein localization, cellular response to stress, and mitotic cell cycle. Moreover, GO term analysis indicated a functional enrichment for respiratory electron-transport chain, nucleotide-excision-repair complex, coated pit, cellular metabolic process, and organic substance transport, among others (Table 2).

Among the 13 interaction loci of TDP-43 tested in the yeast spot assays, 3 genes (SDHA, HSP90AB1, and VDAC3) with strong suppressing effects on TDP-43 toxicity were selected for further evaluation. One gene showing strong suppressing effects was chosen for each group (WT, M337V, and Q331K).

### 3.2. Pharmacological Inhibition of SDHA, HSP90AB1, and VDAC3 Does Not Affect TDP-43-Induced Neurotoxicity

Commercially available pharmacological inhibitors of SDHA (malonate), HSP90AB1 (geldanamycin), and VDAC3 (TRO19622) were used for functional evaluation of the TDP-43 genetic-interacting loci in N2a neuroblastoma cells. N2a is a neuroblastoma cell line that has been used in many TDP-43 studies, particularly for the assessment of TDP-43-induced neurotoxicity and associated molecular mechanisms in vitro [56,57,58]. We first determined the optimal concentration of these pharmacological inhibitors for the N2a cell viability assay (Supplementary Figure S2a). Malonate showed toxicity at 50 and 100 mM, geldanamycin at 5, 50, and 100 µM, and TRO19622 at 5, 50, and 100 µM (Supplementary Figure S2b). With these findings, we were able to determine the optimal concentration of the inhibitors for use in further experiments, i.e., the highest concentration that did not significantly affect cell viability. We investigated the effects of pharmacological inhibition of SDHA, HSP90AB1, and VDAC3 on TDP-43-induced neurotoxicity: N2a cells were first transfected with the control vector, WT, M337V, or Q331K mutant form of human TDP-43 plasmid constructs (all GFP-fused), and the transfected cells were treated with malonate (10 mM), geldanamycin (1 µM), or TRO19622 (1 µM) (Supplementary Figure S3a). The transfection efficiency was confirmed by microscopic evaluation of GFP-positive cells (Supplementary Figure S3b). The results showed that the pharmacological inhibition of SDHA, HSP90AB1, or VDAC3 did not alter TDP-43-induced neurotoxicity (Supplementary Figure S3c). Thus, TDP-43-induced neurotoxicity seems not to be mediated by SDHA, HSP90AB1, or VDAC3 pathways.

### 3.3. Pharmacological Inhibition of SDHA, HSP90AB1, and VDAC3 Attenuates Inflammatory Activation and Mitochondrial Dysfunction of Astrocytes Induced by TDP-43 Overexpression

**Figure 3:**
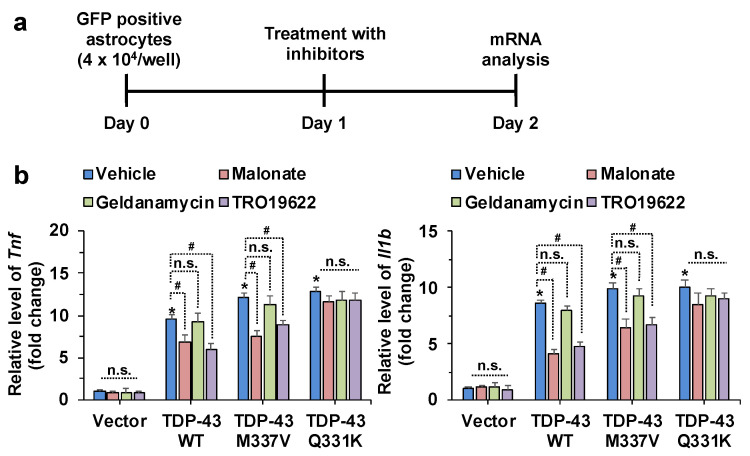
The effects of pharmacological inhibition of SDHA, HSP90AB1, and VDAC3 on the inflammatory activation of astrocytes induced by TDP-43 transfection. (**a**) Astrocytes were transfected with a *GFP*, *TDP-43 WT*, *TDP-43 M337V*, or *TDP-43 Q331K* expression construct. After one day, FACS sorting of GFP-transfected cells was performed. (**b**) The relative levels of *Tnf* and *Il1b* mRNA in FACS-sorted primary astrocytes were assessed by real-time polymerase chain reaction (RT-PCR). Relative gene expression was normalized to the geometric mean of *Gapdh* and *Actb*. * *p* < 0.05 versus vehicle-treated control vector group; # *p* < 0.05 versus indicated groups; n.s., not significant. Data analysis by two-way analysis of variance (ANOVA) with four sister wells (biological replicates); mean ± standard deviation (SD).

We targeted SDHA, HSP90AB1, and VDAC3 using their pharmacological inhibitors (malonate, geldanamycin, and TRO19622, respectively) to investigate the functional role of TDP-43 genetic interaction modifiers in astrocytic phenotypes in vitro. The optimal concentration of inhibitors for primary astrocyte cultures was first determined (Supplementary Figure S4a), and an MTT assay was used to reveal the highest inhibitor concentration that did not significantly affect the cell viability of primary astrocyte cultures (Supplementary Figure S4b). Subsequently, the effects of pharmacological inhibition of SDHA, HSP90AB1, and VDAC3 on TDP-43-induced inflammatory activation of astrocytes were characterized by increased expression of proinflammatory cytokines such as TNF-α and IL-1β at the mRNA and protein levels. The primary astrocytes were first transfected with control vector, WT, or mutant (M337V and Q331K) human TDP-43 plasmid constructs and the transfected cells were treated with malonate (5 mM), geldanamycin (0.5 µM), or TRO19622 (0.5 µM) (Supplementary Figure S5a). The transfection efficiency was confirmed by microscopic evaluation of GFP-positive cells (Supplementary Figure S5b) and Western blot analysis (Supplementary Figure S6). The transfection efficiency of TDP-43 constructs into astrocytes, based on GFP fluorescence, was as follows: GFP vector: 27%, TDP-43 WT: 26%, TDP-43 M337V: 28%, and TDP-43 Q331K: 16% (Supplementary Figure S5b). Fluorescence-activated cell sorting (FACS) of GFP-positive cells was used to isolate TDP-43-transfected cells (Supplementary Figure S7). Transfection with WT and mutant (M337V and Q331K) human TDP-43 was found to similarly increase the expression levels of TNF-α and IL-1β mRNAs (Figure 3 and Supplementary Figure S5c) and proteins (Supplementary Figure S5d) in astrocytes. However, pharmacological inhibition of SDHA and VDAC3 in astrocytes reduced TDP-43 WT or M337V mutant-induced expression levels of TNF-α and IL-1β (Supplementary Figure S5c,d). Notably, the inhibition of HSP90AB1 in astrocytes did not alter TDP-43 WT or M337V mutant-induced expression levels of TNF-α and IL-1β. Similarly, none of the inhibitors significantly influenced Q331K mutant-induced inflammatory cytokines. These findings imply that SDHA and VDAC3 play critical roles in TDP-43 WT or M337V mutant-induced inflammatory activation of astrocytes (Supplementary Figure S5c,d).

**Figure 4:**
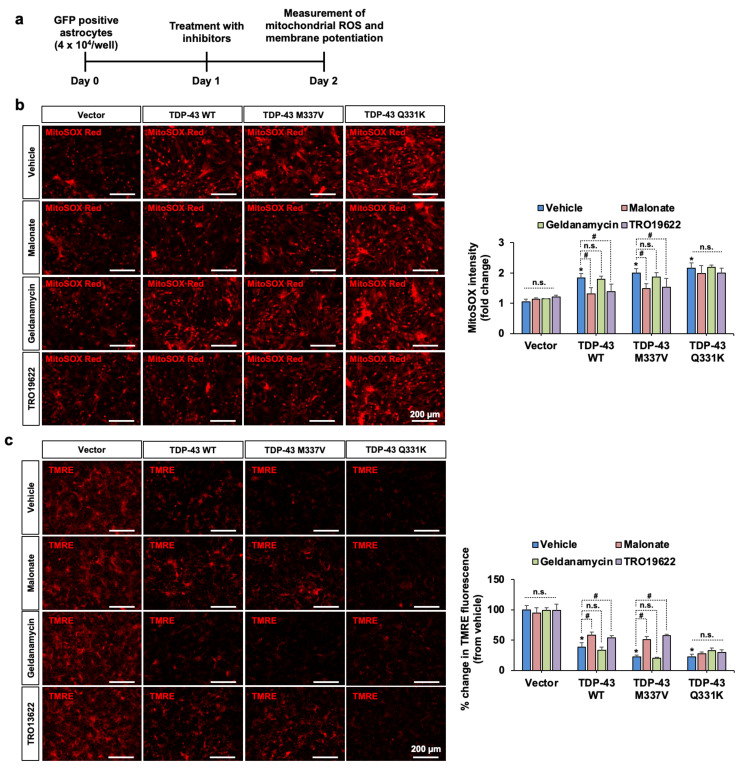
The effects of pharmacological inhibition of SDHA, VDAC3, and HSP90AB1 on TDP-43 WT and mutant-induced mitochondrial dysfunction in primary astrocytes. (**a**) Experimental timeline. (**b**) Mitochondrial reactive oxygen species (ROS) was measured by MitoSOX staining. The fluorescence intensity of MitoSOX is shown as fold change in the adjacent graph. (**c**) Astrocytic mitochondrial membrane potential was assessed by TMRE probing. Percent reduction in TMRE fluorescence is shown in the adjacent graph. Scale bars indicate 200 µm. * *p* < 0.05 versus vehicle-treated control vector group; # *p* < 0.05 versus indicated groups; n.s., not significant. Data analysis by two-way ANOVA with four sister wells (biological replicates); mean ± SD.

Finally, we investigated the effects of TDP-43 and pharmacological inhibition of SDHA, HSP90AB1, and VDAC3 on mitochondrial membrane potential and ROS levels in astrocytes. Transfection of astrocytes with WT and mutant (M337V and Q331K) TDP-43 was found to reduce mitochondrial membrane potential and increase levels of mitochondrial ROS in astrocytes (Figure 4). Pharmacological inhibition of SDHA and VDAC3, but not HSP90AB1, in astrocytes ameliorated TDP-43 WT or M337V mutant-induced impairment of astrocytic mitochondrial functions. None of the inhibitors significantly influenced Q331K mutant-induced mitochondrial dysfunction. These findings imply that both SDHA and VDAC3 are involved in TDP-43-induced mitochondrial dysfunction and the subsequent inflammatory activation of astrocytes.

## 4. Discussion

Our findings from *TDP-43* genetic interaction analyses and pharmacological validation experiments indicate that *TDP-43* and its interactors *SDHA* and *VDAC3* play important roles in the inflammatory activation of astrocytes; consequently, they may also be associated with the pathological features of ALS and FTD.

A large-scale human–yeast genetic interaction screen was previously performed to elucidate the molecular pathways of online mendelian inheritance in man (OMIM) genes [33] and protein kinases [32]. OMIM is a compendium of human genes and genetic phenotypes for all known Mendelian disorders. OMIM focuses on the relationship between phenotype and genotype [59,60]. Unfortunately, however, TDP-43 was not included in the initial human open reading frame clone (ORFeome) collections used for screen. Future study is required to elucidate the significance of kinase-mediated modification of TDP-43. A similar approach was applied to the *TDP-43* gene in this study. Transformation of human *TDP-43* cDNAs of WT or disease-associated mutants (*M337V* and *Q331K*) into a 4653 homozygous diploid yeast deletion set and subsequent barcode sequencing identified 13 yeast toxicity suppressors with human orthologs. Our findings reveal a multitude of known co-localization and co-expression genes, as well as novel *TDP-43* genetic interaction loci and related genes that warrant further study. In our network analysis, co-expression of genes only indicates that they could be functional at the same time or in the same space—it does not imply functional linkage, they may function antagonistically or collaboratively for the same process or they could be functioning in two completely unrelated processes. Thus, the co-expression or co-localization in the current network require further investigation to demonstrate a functional relationship. GO analysis of *TDP-43* genetic interaction loci has demonstrated that there is an enrichment of genes related to cellular metabolic process (Table 2, Figure 2). Supporting our data, a recent study using proteomics and bioinformatics tools has reported activation of stress responses in gastrocnemius muscle of ALS-Tg mice, involving HSP90AB1, abnormalities in the endoplasmic reticulum (ER) protein folding machinery, and activation of the unfolded protein response (UPR) [61]. Mutations in PEX12 have been recently correlated with progressive neurological disorders [62,63]. Lipid metabolism defects have also been found in both AD and PD. This evidence suggests that peroxisome biogenesis may play an important role in aging and associated disorders. Interestingly, high levels of succinate and the induction of Hypoxia-inducible factor 1-α (HIF-1α) were observed in SDHA-deficient fibroblasts [64]. Bi-allelic mutations of SDHA were described in Leigh syndrome, an early-onset, progressive neurodegenerative disease caused by defective mitochondrial bioenergetics [65]. Although the current *TDP-43* interaction network was limited to the 13 toxicity modifiers and their related genes, our results provide a platform on which to construct a broader network of *TDP-43* genetic interactions in the future. The TDP-43 network identified in the current study also improves our understanding of the TDP-43 signaling pathways relevant to both the healthy and diseased brain.

TDP-43 has previously been implicated in various aspects of the mRNA life cycle [66], cell toxicity [67], and neuroinflammation [22]. A recent study has shown the interaction between TDP-43 and human elongation factor for RNA polymerase 2 (ELL2) by co-immunoprecipitation from human cells. These findings reveal important roles of ELL complexes little elongation complex (LEC) and super elongation complex (SEC) in TDP-43-associated toxicity, providing potential therapeutic insight for TDP-43-associated neurodegeneration [68]. Our analyses of *TDP-43* genetic interactions revealed a global view of *TDP-43* pathways, some of which are involved in cellular metabolic processes and organic substance transport. We also found that *SDHA*, *HSP90AB1*, and *VDAC3* are the major interacting genetic loci of *TDP-43*, and these three interacting loci showed strong toxicity-suppressing effects and were therefore further investigated in relation to their inflammatory activation of astrocytes and neurotoxicity. Previously, it was reported that both neurons [69,70,71] and glia (microglia and astrocytes) [22,72,73] show TDP-43-mediated proteinopathy and subsequent motor neuronal death associated with ALS and FTD. The timeframe of onset of ALS-associated TDP-43 proteinopathy has been classified into four stages by Brettschneider and Braak et al. [74,75]: in stage 1, p-TDP-43 proteinopathy mainly occurs in the projection neurons of the agranular motor cortex and in the somatomotor neurons of the brainstem and spinal cord; in stage 2, proteinopathy is observed in the prefrontal cortex, precerebellar nuclei of the brainstem, reticular formation, and parvocellular portions of the red nucleus; in stage 3, proteinopathy develops in the prefrontal cortex, striatum, and basal ganglia; finally, in stage 4, p-TDP-43 proteinopathy extensively progresses into the anteromedial areas of the temporal lobe, the entorhinal cortex, and the hippocampus [76,77,78]. A previous study has shown that the expression of human TDP-43 (hTDP-43) harboring a defective nuclear localization signal (ΔNLS) results in the aggregation of insoluble, phosphorylated cytoplasmic TDP-43 in the brain and spinal cord, loss of endogenous nuclear mouse TDP-43 (mTDP-43), brain atrophy, muscle denervation, dramatic motor neuron loss, and progressive motor impairments [69]. Interestingly, hTDP-43-ΔNLS expression did not alter the number of spinal motor neurons at the lumbar L4–L5 level at the four-week time point but was reduced by 28% after six weeks; by eight weeks of hTDP-43-ΔNLS expression, only 50% of lumbar motor neurons remained.

Recent findings, particularly in ALS models [18,79], have demonstrated that glial pathology may play a critical role in disease progression, as characterized by the upregulation of diverse inflammatory molecules [80]. A study by Serio et al. demonstrated the existence of a cell-autonomous pathological phenotype in human astrocytes derived from patients with ALS-causing TDP-43 mutations [81]. A previous study also showed that reactive astrocytes from patients with ALS are toxic to motor neurons during coculture [82]. Recently, Qian et al. reported that astrocytes from patients with ALS cause neurodegeneration and movement deficits in mice, following their transplantation into the spinal cord [83]. Movement deficits (assessed according to the locomotor behaviors of mouse forelimbs) were observed at six and nine months post-cell transplantation, with neurodegeneration phenotypes being observed after nine months post-cell transplantation. Although these studies do not provide a clear molecular mechanism of astrocyte-mediated neurotoxicity in ALS, a recent study by Lee et al. revealed that the overexpression of *TDP-43* in astrocytes induces neurodegeneration via a PTP1B-mediated inflammatory response [22]. Nevertheless, the possibility remains that other molecules are involved in the TDP-43 pathway in astrocytes.

Cytoplasmic aggregation of TDP-43 is a common phenotype observed in majority of ALS cases [84] and has been associated with neurotoxicity of motor neurons. Besides transcription repressor activity of TDP-43, various other biological functions of TDP-43 have been identified, including mitochondrial function and metabolism [85]. Recent literature advocates the role of TDP-43 cytoplasmic inclusion in astrocytes causing inflammatory activation and exacerbating neurotoxicity in ALS pathology [8]. Inflammatory activation of astrocytes induced by TDP-43 inclusions involves various mechanisms, one of which includes alteration of metabolic machinery of astrocytes. It has been reported recently that overexpression of mutated TDP-43 in rat cortical astrocytes results in heightened accumulation of lipid droplets and increased aerobic glycolysis, leading to impaired neuronal metabolic support [86]. In line with previous reports, our results also confirm the mitochondrial dysfunction induced by TDP-43 inclusions in astrocytes. Our genetic screening results identified SDHA and VDAC3 as strong interaction loci of TDP-43, and their pharmacological inhibition in TDP-43-bearing astrocytes reduced inflammatory activation of astrocytes, seconding the potential role of TDP-43 in altering mitochondrial integrity and henceforth, metabolism.

Our study demonstrates that pharmacological inhibition of SDHA and VDAC3 substantially attenuates WT or M337V mutant TDP-43-induced inflammatory activation of astrocytes, as characterized by the expression of TNF-α and IL-1β. However, inhibition of SDHA or VDAC3 does not alter Q331K mutant-induced astrocytic activation. We, therefore, suggest that WT or M337V mutant TDP-43-induced inflammatory activation of astrocytes is mediated by SDHA or VDAC3 pathways, and that they potentially play roles in neuroinflammation associated with the pathogenesis of ALS [18,22,83]. However, results from another mutant Q331K indicate that SDHA or VDAC3 may not always be involved in inflammatory activation of astrocytes. TDP-43 WT and M337V mutant-overexpressing mouse models are reported to show similarities in disease phenotypes, including gait impairment, neurodegeneration, and TDP-43 misprocessing [87]. A recent study compared the effects of the independent expression of TDP-43 WT and the expression of the Q331K mutant [88]. TDP-43 WT expression did not induce any clinical or pathological phenotypes, whereas the Q331K mutant expression induced cytoplasmic accumulation of TDP-43 along with neuronal loss and glial activation in the spinal cord and motor cortex. The molecular mechanisms underlying the differential effects of these TDP-43 genetic loci should be further investigated in the future. In agreement with our findings, both SDHA [89] and VDAC [90] are known to play important roles in disease-associated inflammatory activation of immune cells, including microglia and macrophages. As a possible mechanism, Mills et al. reported that SDHA supports metabolic repurposing of mitochondria and the production of ROS to drive inflammatory phenotypes of macrophages [89,91]. Similarly, a VDAC dimer has been reported to increase ROS production, as well as nucleotide oligomerization domain (NOD)-like receptor family pyrin domain containing 3 (NLRP3) and IL-1β levels in the mouse brain with intracerebral hemorrhage [90].

## 5. Conclusions

Our studies found 13 novel genetic interaction loci of *TDP-43* showing suppressive effects on TDP-43 toxicity, which have not been reported previously. Functional analysis provides evidence for the role of these TDP-43-interacting loci, especially *SDHA* and *VDAC3*, in inflammatory activation of astrocytes and concurrent neuroinflammation in the pathogenesis of ALS and FTD.

## Figures and Tables

**Table 1:** List of TDP-43 genetic interaction candidates tested in the yeast spot assays.

Geno-Type	No.	Yeast Gene Names		UniProt ID	Description	Human Orthologs
TDP-43 WT	1	YCR011C	*ADP1*	P25371	Probable ATP-dependent permease	*ABCG2*
	2	YLR172C	*DPH5*	P32469	Diphthine methyl ester synthase	*DPH5*
	3	YJL134W	*LCB3*	P47013	Dihydrosphingosine 1-phosphate phosphatase LCB3	*SGPP1*
	4	YBL057C	*PTH2*	P34222	Peptidyl-tRNA hydrolase 2	*DPP4*
	5	YIL064W	*EFM4*	P40516	Protein-lysine N-methyltransferase EFM4	*METTL10*
	6	YJL045W	*SDH*	P47052	Succinate dehydrogenase [ubiquinone] flavoprotein subunit 2, mitochondrial	*SDHA*
	7	YJR058C	*APS2*	Q00381	AP-2 complex subunit sigma	*AP2S1*
	8	YML042W	*CAT2*	P32796	Carnitine O-acetyltransferase, mitochondrial	*CRAT*
	9	YER177W	*BMH1*	P29311	Protein BMH1	*YWHAE*
	10	YMR118C	*SHH3*	Q04487	Mitochondrial inner membrane protein SHH3	*SDHC*
TDP-43 M337V	1	YMR002W	*MIX17*	Q03667	Mitochondrial intermembrane space cysteine motif-containing protein MIX17	*CHCHD2*
	2	YMR242C	*RPL20A*	P0CX23	60S ribosomal protein L20-A	*RPL18A*
	3	YPL195W	*APL5*	Q08951	AP-3 complex subunit delta	*AP3D1*
	4	YER005W	*YND1*	P40009	Golgi apyrase	*ENTPD7*
	5	YEL020C	*PXP1*	P39994	Putative 2-hydroxyacyl-CoA lyase	*HACL1*
	6	YJR148W	*BAT2*	P47176	Branched-chain-amino-acid aminotransferase, cytosolic	*BCAT1*
	7	YIL114C	*POR2*	P40478	Mitochondrial outer membrane protein porin 2	*VDAC3*
	8	YIL160C	*POT1*	P27796	3-ketoacyl-CoA thiolase, peroxisomal	*ACAA1*
	9	YMR118C	*SHH3*	Q04487	Mitochondrial inner membrane protein SHH3	*SDHC*
	10	YMR035W	*IMP2*	P46972	Mitochondrial inner membrane protease subunit 2	*IMMP2L*
TDP-43 Q331K	1	YAL023C	*PMT2*	P31382	Dolichyl-phosphate-mannose--protein mannosyltransferase 2	*MTHFR*
	2	YAR042W	*SWH1*	P35845	Oxysterol-binding protein homolog 1	*OSBP*
	3	YLR059C	*REX2*	P54964	Oligoribonuclease, mitochondrial	*REXO2*
	4	YMR026C	*PEX12*	Q04370	Peroxisome assembly protein 12	*PEX12*
	5	YML087C	*AIM33*	Q04516	Uncharacterized oxidoreductase AIM33	*CYB5R1*
	6	YMR186W	*HSC82*	P15108	ATP-dependent molecular chaperone HSC82	*HSP90AB1*
	7	YOR002W	*ALG6*	Q12001	Dolichyl pyrophosphate Man9GlcNAc2 alpha-1,3-glucosyltransferase	*ALG6*
	8	YOR089C	*VPS21*	P36017	Vacuolar protein sorting-associated protein 21	*RAB5A*
	9	YPL207W	*TYW1*	Q08960	S-adenosyl-L-methionine-dependent tRNA 4-demethylwyosine synthase	*TYW1*
	10	YOL080C	*REX4*	Q08237	RNA exonuclease 4	*AEN*
	11	YPL198W	*RPL7B*	Q12213	60S ribosomal protein L7-B	*RPL7*
	12	YPL101W	*ELP4*	Q02884	Elongator complex protein 4	*ELP4*
	13	YBR241C	*YBR241C*	P38142	Probable metabolite transport protein YBR241C	*SLC2A1*
	14	YEL024W	*RIP1*	P08067	Cytochrome b-c1 complex subunit Rieske, mitochondrial	*UQCRFS1*
	15	YEL053C	*MAK10*	Q02197	N-alpha-acetyltransferase 35, NatC auxiliary subunit	*NAA35*
	16	YHR161C	*YAP1801*	P38856	Clathrin coat assembly protein AP180A	*PICALM*
	17	YCL008C	*STP22*	P25604	Suppressor protein STP22 of temperature-sensitive alpha-factor receptor and arginine permease	*TSG101*
	18	YLR209C	*PNP1*	Q05788	Purine nucleoside phosphorylase	*PNP*
	19	YOR120W	*GCY1*	P14065	Glycerol 2-dehydrogenase (NADP(+))	*AKR1A1*
	20	YPL088W	*AAD16*	Q02895	Putative aryl-alcohol dehydrogenase AAD16	*KCNAB1*
	21	YNL155W	*CUZ1*	P53899	CDC48-associated ubiquitin-like/zinc finger protein 1	*ZFAND1*
	22	YIL044C	*AGE2*	P40529	ADP-ribosylation factor GTPase-activating protein effector protein 2	*SMAP2*
	23	YIL079C	*AIR1*	P40507	Protein AIR1	*ZCCHC7*
	24	YIL076W	*SEC28*	P40509	Coatomer subunit epsilon	*COPE*
	25	YER095W	*RAD51*	P25454	DNA repair protein RAD51	*RAD51*
	26	YJL065C	*DLS1*	P40366	Protein DLS1	*CHRAC1*
	27	YJL121C	*RPE1*	P46969	Ribulose-phosphate 3-epimerase	*RPE*
	28	YJR035W	*RAD26*	P40352	DNA repair and recombination protein RAD26	*ERCC6*
	29	YIL007C	*NAS2*	P40555	Probable 26S proteasome regulatory subunit p27	*PSMD9*
	30	YNL130C	*CPT1*	P17898	Cholinephosphotransferase 1	*SELENOI*
	31	YDL022W	*GPD1*	Q00055	Glycerol-3-phosphate dehydrogenase [NAD(+)] 1	*GPD1*
	32	YDL036C	*PUS9*	Q12069	tRNA pseudouridine(32) synthase, mitochondrial	*RPUSD2*
	33	YPL013C	*MRPS16*	Q02608	37S ribosomal protein S16, mitochondrial	*MRPS16*

**Table 2:** The gene ontology (GO) terms enriched in the *TDP-43* network constructed in this study.

GO Term	Genes	Q-Value ^a^
Respiratory electron transport chain	*CYCS, **SDHA**, SDHAF2, SDHB, SDHC, SDHD*	2.27 × 10^−4^
Nucleotide-excision repair complex	***CHRAC1*** *, ERCC8, POLE3*	3.78 × 10^−3^
Coated pit	*CLTC, CLTCL1, EPS15, **PICALM***	1.16 × 10^−2^
Cellular metabolic process	***HSP90AB1*** *, **ERCC6**, **SDHA**, **CHRAC1**, **SELENOI**, **PICALM**, **NAA35**, **OSBP**, **PEX12**, **METTL10**, **PNP**, **AEN**, IKBKE, ANKZF1*	3.39 × 10^−2^
Protein C-terminus binding	***ERCC6*** *, PEX10, **PEX12**, PEX5*	3.83 × 10^−2^
Protein serine/threonine phosphatase activity	*CYCS, PPFIA1, PPP5C*	3.95 × 10^−2^
Organic substance transport	***OSBP*** *, **PNP**, **VDAC3**, ATP10D, PEX10, **PEX12***	4.46 × 10^−2^
Nucleotide-binding domain, leucine-rich repeat-containing receptor signaling pathway	***HSP90AB1*** *, NLRP3, SUGT1*	5.31 × 10^−2^

Note: Bold entries indicate the genetic interaction loci of *TDP-43* identified in this study. **^a^** Q-values are estimates obtained using the Benjamin-Hochberg procedure.

## Data Availability

The dataset supporting the conclusions of this article is included within the article and its additional file.

## Supplementary Materials

The following are available online at https://www.mdpi.com/2073-4409/10/3/676/s1, Table S1: Barcode counts of yeast deletion strains obtained from high-throughput sequencing. Table S2: The list of overlapping genes. Figure S1: Expression of GFP-tagged-*TDP-43 WT*, *TDP-43 M337V*, and *TDP-43 Q331K* in yeast. Representative images and quantification of GFP vector, TDP-43 WT, TDP-43 M337V, and TDP-43 Q331K (*n* = 4). Scale bar: 100 μm. AU, arbitrary unit. Data represented as mean ± SD. Figure S2: Determination of the optimal concentration of pharmacological inhibitors for the neurotoxicity experiments. (a) Diagram showing the timeline of experimentation. (b) N2a neuroblastoma cells were treated with increasing concentrations of malonate, geldanamycin, or TRO19622, and cell viabilities were then assessed with an MTT assay. * *p* < 0.05 versus vehicle-treated group. Student’s t-test (control versus each treatment condition) on eight sister wells (biological replicates); data are mean ± SD. Figure S3: The effects of pharmacological inhibition of SDHA, HSP90AB1, and VDAC3 on TDP-43-induced neurotoxicity. (a) Diagram showing the experimentation timeline. (b) Microscopic data show the GFP expression indicating the transfection efficiency of the control vector, *TDP-43 WT*, *TDP-43 M337V*, and *TDP-43 Q331K* in N2a cells. Quantification of the proportion of GFP-positive cells for the control vector, *TDP-43 WT*, *TDP-43 M337V*, and *TDP-43 Q331K*. Graph shows the mean ± SD (*n* = 4). (c) N2a cell viability was assessed after *TDP-43 WT*, *TDP-43 M337V*, and *TDP-43 Q331K* transfection followed by malonate (10 mM), geldanamycin (1 µM), and TRO19622 (1 µM) treatment. * *p* < 0.05 versus vehicle-treated control vector group; n.s., not significant; two-way ANOVA on eight sister wells (biological replicates); data are mean ± SD. Figure S4: Determination of the optimal concentration of pharmacological inhibitors for primary astrocyte cultures. (a) Diagram showing the timeline of experimentation. (b) Primary astrocytes were treated with increasing concentrations of malonate, geldanamycin, or TRO19622, and cell viabilities were then assessed with an MTT assay. * *p* < 0.05 versus vehicle-treated group; Student’s t-test (control versus each treatment condition) on eight sister wells in culture plates (*n* = 8); data are mean ± SD. Figure S5: The effects of pharmacological inhibition of SDHA, HSP90AB1, and VDAC3 on the inflammatory activation of astrocytes induced by *TDP-43* transfection. (a) Diagram showing the timeline of experimentation. (b) Microscopic data show GFP expressions indicating the transfection efficiency of the control vector, *TDP-43 WT*, *TDP-43 M337V*, and *TDP-43 Q331K* in primary astrocytes. Quantification of the proportion of GFP-positive cells for the control vector, TDP-43 WT, TDP-43 M337V, and TDP-43 Q331K. The graph shows the mean ± SD (*n* = 4). (c and d) The relative levels of Tnf and Il1b mRNA (c) and protein (d) in primary astrocytes were assessed by RT-PCR and ELISA respectively, after *TDP-43 WT*, *TDP-43 M337V*, or *TDP-43 Q331K* transfection, followed by malonate (5 mM), geldanamycin (0.5 µM), or TRO19622 (0.5 µM) treatment. Relative gene expression was normalized to the geometric mean of *Gapdh* and *Actb*. * *p* < 0.05 versus vehicle-treated control vector group; # *p* < 0.05 versus indicated groups; n.s., not significant; two-way ANOVA on six sister wells in culture plates (*n* = 6); data are mean ± SD. Figure S6: Western blot detection of TDP-43 in astrocytes. (a) Experimental timeline. (b) Protein levels of TDP-43 were assessed by Western blot in the primary astrocyte cultures transfected with *GFP vector*, *TDP-43 WT*, *TDP-43 M337V*, or *TDP-43 Q331K*. Figure S7: FACS sorting of GFP-positive astrocytes. (a) Experimental timeline. (b) Astrocytes were transiently transfected with *GFP* vector, *TDP-43 WT*, *TDP-43 M337V*, or *TDP-43 Q331K* plasmids. GFP-positive cells were isolated by FACS sorting. GFP or GFP-fused TDP-43 expression in the FACS-sorted cells was detected under a fluorescence microscope. Scale bar: 200 μm.
