# Supplementary material for: Identification of Genetic Modifiers of TDP-43: Inflammatory Activation of Astrocytes for Neuroinflammation
Source: Cells. 2021 Mar 18;10(3):676. doi: 10.3390/cells10030676 (PMC8003223; doi:10.3390/cells10030676)
Supplement: Supplementary file 1 [file cells-10-00676-s001.zip › Supplementary Figure 1.pdf]

Supplementary Figure 1

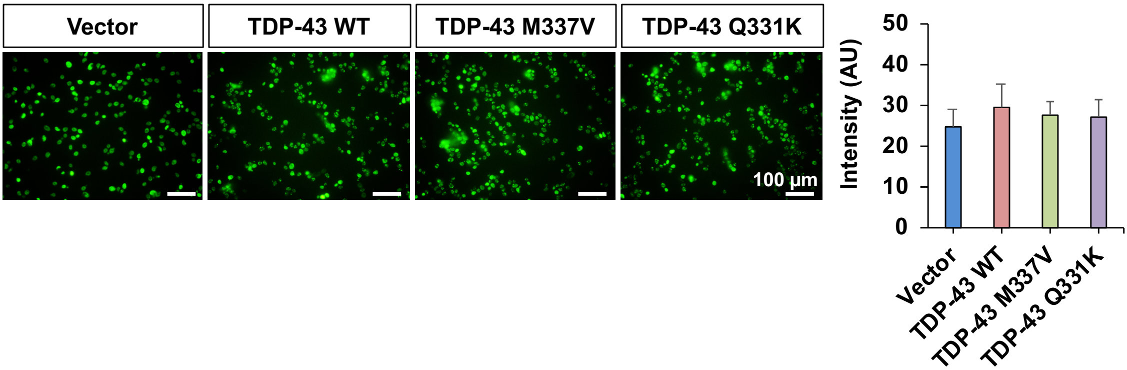

Supplementary Figure 1. Expression of GFP-tagged-TDP-43 WT, TDP-43 M337V, and TDP-43 Q331K in yeast. Representative images showing the GFP signals after transfecting yeast with *TDP-43 WT*, *TDP-43 M337V*, and *TDP-43 Q331K* (n = 4). Quantification of GFP intensity in each group is shown in the adjacent graph. Scale bar, 100 μm. AU, arbitrary units. Data are represented as mean ± SD.
