# Supplementary material for: Identification of Genetic Modifiers of TDP-43: Inflammatory Activation of Astrocytes for Neuroinflammation
Source: Cells. 2021 Mar 18;10(3):676. doi: 10.3390/cells10030676 (PMC8003223; doi:10.3390/cells10030676)
Supplement: Supplementary file 1 [file cells-10-00676-s001.zip › Supplementary Figure 2.pdf]

**Supplementary Figure 2**

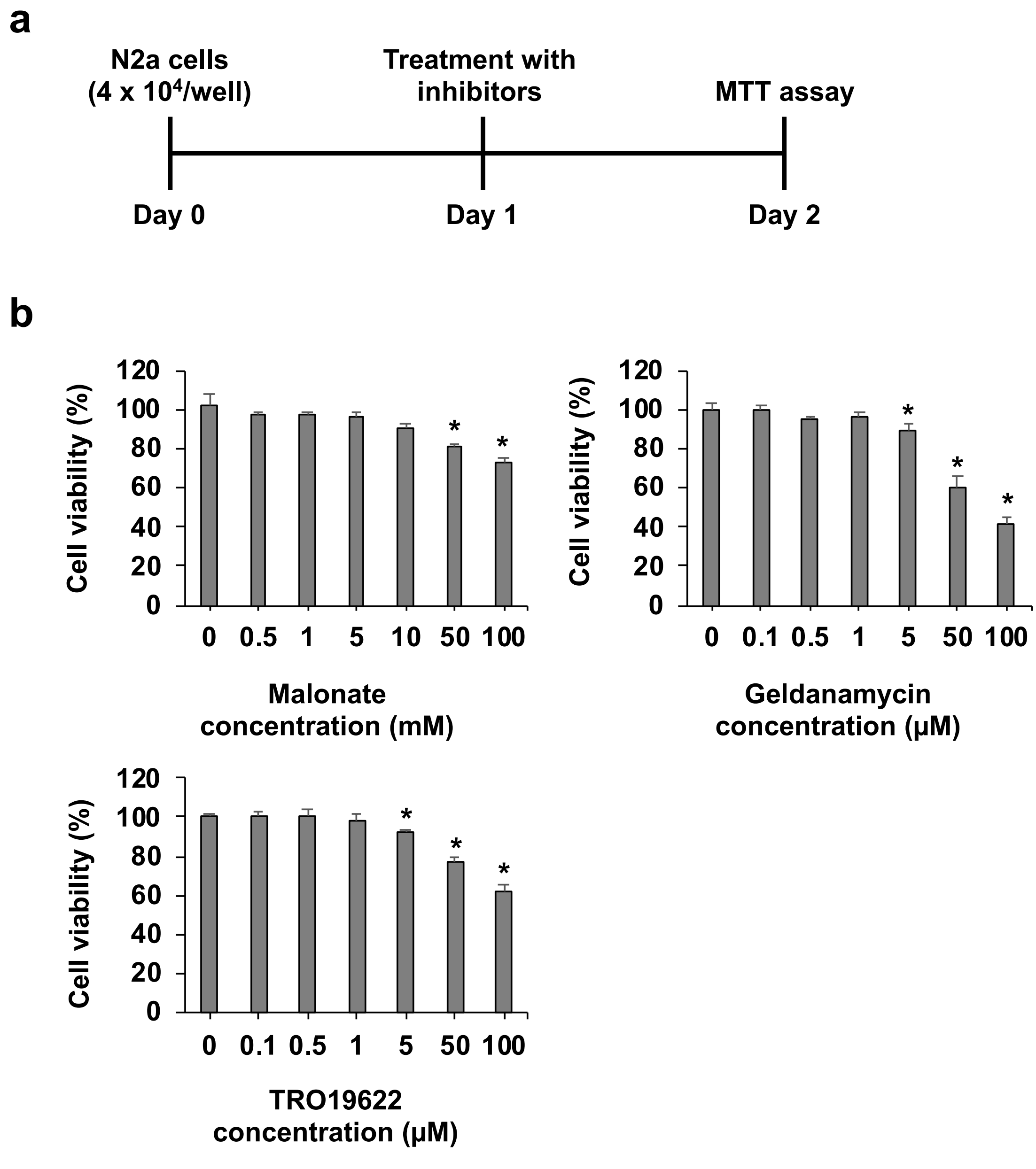


**Supplementary Figure 2.** Determination of optimal concentration of pharmacological inhibitors for the neurotoxicity experiments. (**a**) The diagram shows the timeline of experimentation. (**b**) The N2a neuroblastoma cells were treated with increasing concentrations of malonate, geldanamycin, or TRO19622, and the cell viabilities were then assessed by MTT assay. *P < 0.05 versus vehicle-treated group. Student's t-test (control versus each treatment condition) and eight sister wells (biological replicates); mean ± SD.
