# Supplementary material for: Identification of Genetic Modifiers of TDP-43: Inflammatory Activation of Astrocytes for Neuroinflammation
Source: Cells. 2021 Mar 18;10(3):676. doi: 10.3390/cells10030676 (PMC8003223; doi:10.3390/cells10030676)
Supplement: Supplementary file 1 [file cells-10-00676-s001.zip › Supplementary Figure 3.pdf]

Supplementary Figure 3

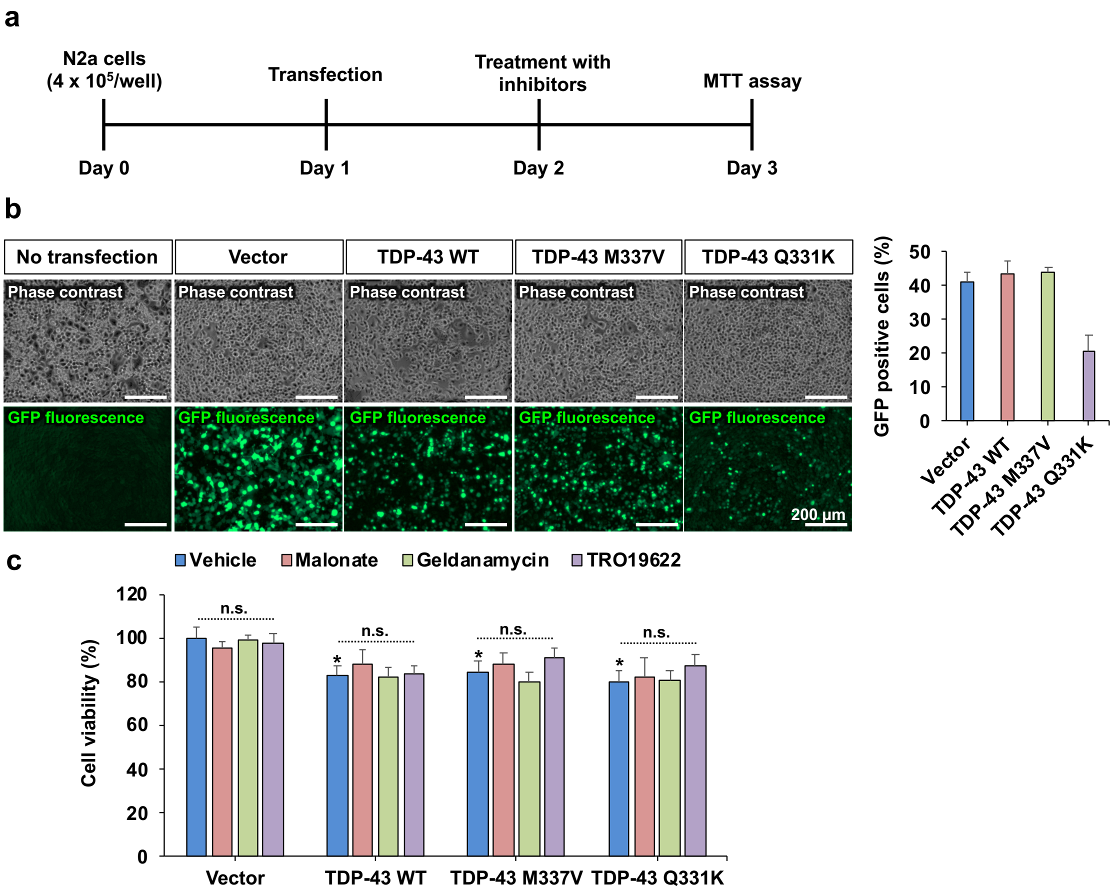

Supplementary Figure 3. The effects of pharmacological inhibition of SDHA, HSP90AB1, and VDAC3 on TDP-43-induced neurotoxicity. (**a**) The diagram shows the timeline of experimentation. (**b**) Microscopic data show the GFP expression indicating transfection efficiency of the control vector, *TDP-43 WT*, *TDP-43 M337V*, and *TDP-43 Q331K* in N2a cells. Quantification of percent of GFP-positive cells for control vector, TDP-43 WT, TDP-43 M337V, and TDP-43 Q331K. Graph shows the mean ± SD (n=4). (**c**) The N2a cell viability was assessed after *TDP-43 WT*, *TDP-43 M337V*, and *TDP-43 Q331K* transfection followed by malonate (10 mM), geldanamycin (1 µM), and TRO19622 (1 µM) treatment. *P < 0.05 versus vehicle-treated control vector group; n.s., not significant. Two-way ANOVA and eight sister wells (biological replicates); mean ± SD.
