# Supplementary material for: Identification of Genetic Modifiers of TDP-43: Inflammatory Activation of Astrocytes for Neuroinflammation
Source: Cells. 2021 Mar 18;10(3):676. doi: 10.3390/cells10030676 (PMC8003223; doi:10.3390/cells10030676)
Supplement: Supplementary file 1 [file cells-10-00676-s001.zip › Supplementary Figure 4.pdf]

Supplementary Figure 4

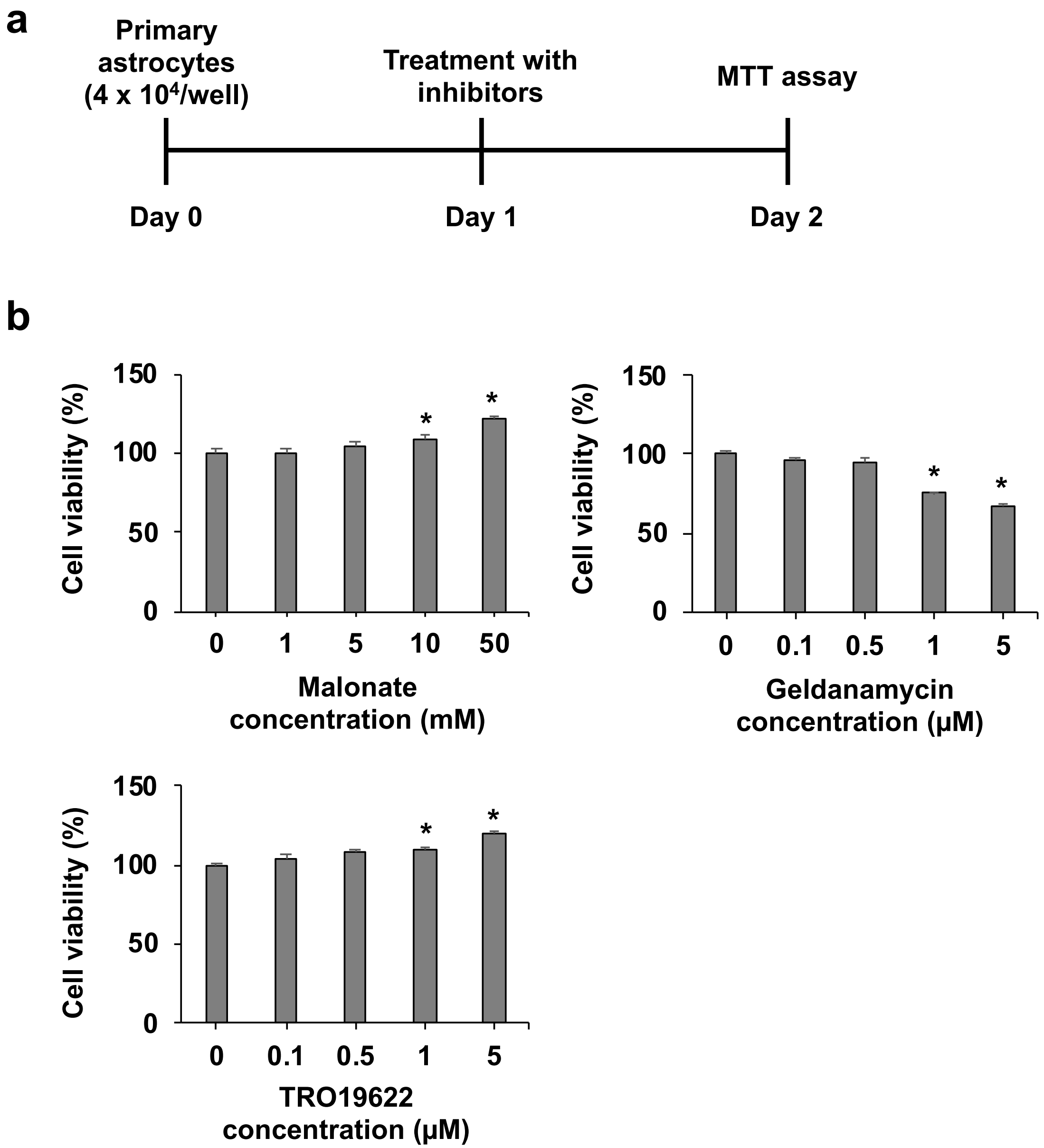

Supplementary Figure 4. Determination of optimal concentration of pharmacological inhibitors for primary astrocyte cultures. (**a**) The diagram shows the timeline of experimentation. (**b**) Primary astrocytes were treated with increasing concentrations of malonate, geldanamycin, or TRO19622; cell viabilities were then assessed by MTT assay. *P < 0.05 versus vehicle-treated group. Student's t-test (control versus each treatment condition) and n=8 sister wells in culture plates; mean ± SD.
