# Supplementary material for: Identification of Genetic Modifiers of TDP-43: Inflammatory Activation of Astrocytes for Neuroinflammation
Source: Cells. 2021 Mar 18;10(3):676. doi: 10.3390/cells10030676 (PMC8003223; doi:10.3390/cells10030676)
Supplement: Supplementary file 1 [file cells-10-00676-s001.zip › Supplementary Figure 5.pdf]

Supplementary Figure 5

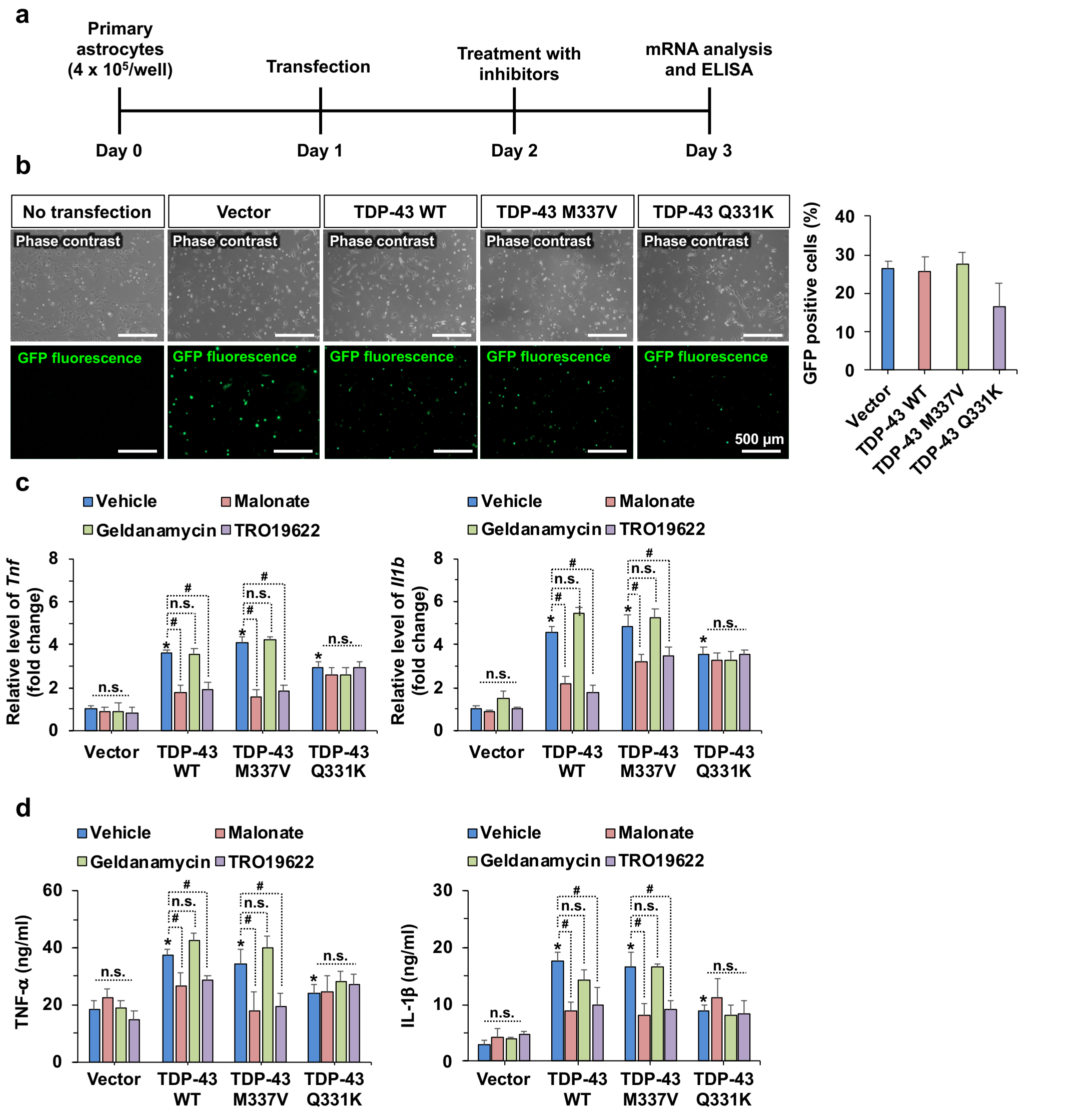

Supplementary Figure 5. The effects of pharmacological inhibition of SDHA, HSP90AB1, and VDAC3 on the inflammatory activation of astrocytes induced by *TDP-43* transfection. (**a**) The diagram shows the timeline of experimentation. (**b**) Microscopic data show the GFP expressions indicating transfection efficiency of the control vector, *TDP-43 WT*, *TDP-43 M337V*, and *TDP-43 Q331K* in primary astrocytes. Quantification of percent of GFP-positive cells for control vector, TDP-43 WT, TDP-43 M337V, and TDP-43 Q331K. Graph shows the mean ± SD (n=4). (c and d) The relative levels of *Tnf* and *Il1b* mRNA (**c**) and protein (**d**) in primary astrocytes were assessed by real time-RT PCR and ELISA, respectively, after *TDP-43 WT*, *TDP-43 M337V*, or *TDP-43 Q331K* transfection followed by malonate (5 mM), geldanamycin (0.5 μM), or TRO19622 (0.5 μM) treatment. Relative gene expression was normalized to the geometric mean of *Gapdh* and *Actb*. *P < 0.05 versus vehicle-treated control vector group; #P < 0.05 versus indicated groups; n.s., not significant. Two-way ANOVA and n=6 sister wells in culture plates; mean ± SD.
