# Supplementary material for: Identification of Genetic Modifiers of TDP-43: Inflammatory Activation of Astrocytes for Neuroinflammation
Source: Cells. 2021 Mar 18;10(3):676. doi: 10.3390/cells10030676 (PMC8003223; doi:10.3390/cells10030676)
Supplement: Supplementary file 1 [file cells-10-00676-s001.zip › Supplementary Figure 6.pdf]

Supplementary Figure 6

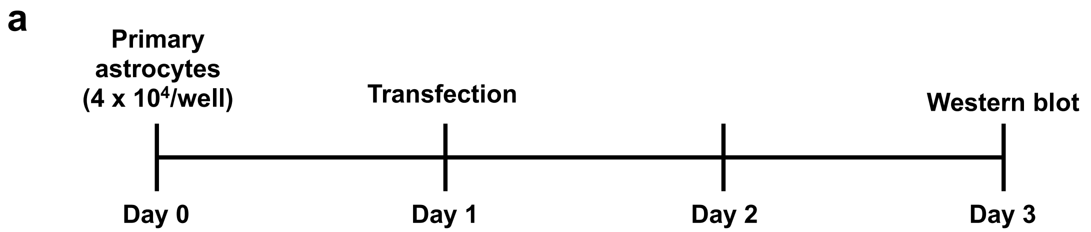

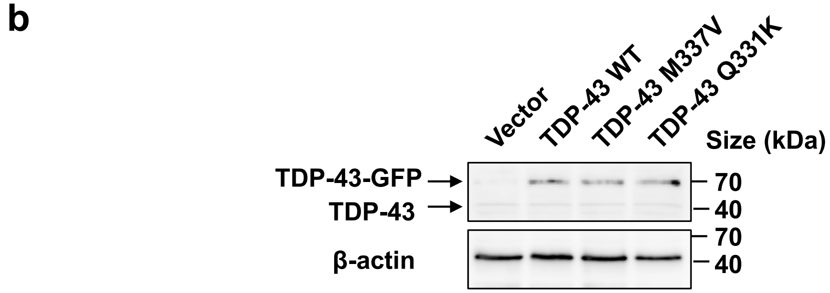

Supplementary Figure 6. Western blot detection of TDP-43 in astrocytes. (**a**) Experimental timeline. (**b**) The protein levels of TDP-43 were assessed by western blot in the primary astrocyte cultures transfected with *GFP* vector, *TDP-43 WT*, *TDP-43 M337V*, or *TDP-43 Q331K*.
