# Supplementary material for: Identification of Genetic Modifiers of TDP-43: Inflammatory Activation of Astrocytes for Neuroinflammation
Source: Cells. 2021 Mar 18;10(3):676. doi: 10.3390/cells10030676 (PMC8003223; doi:10.3390/cells10030676)
Supplement: Supplementary file 1 [file cells-10-00676-s001.zip › Supplementary Table 2.docx]

**Table S2. The list of overlapping genes.**

| **No.** | **Overlapping gene names** | **Description** | **References** |
| --- | --- | --- | --- |
| 1 | RPL18A | 60S ribosomal protein L18a | [1] |
| 2 | RPL7 | 60S ribosomal protein L7 | [1] |
| 3 | OSBP | Oxysterol-binding protein 1 | [2] |
| 4 | KCNAB3 | Voltage-gated potassium channel subunit beta-3 | [2] |
| 5 | REXO2 | Oligoribonuclease, mitochondrial | [3] |
| 6 | PICALM | Phosphatidylinositol-binding clathrin assembly protein | [3] |
